# Incidence and timing of potentially high-risk arrhythmias detected through long term continuous ambulatory electrocardiographic monitoring

**DOI:** 10.1186/s12872-016-0210-x

**Published:** 2016-02-17

**Authors:** Matthew D. Solomon, Jingrong Yang, Sue Hee Sung, Martha L. Livingston, George Sarlas, Judith C. Lenane, Alan S. Go

**Affiliations:** Division of Research, Kaiser Permanente Northern California, Oakland, CA USA; Stanford University School of Medicine, Stanford, CA USA; iRhythm Technologies, Inc, San Francisco, CA USA; Departments of Epidemiology, Biostatistics and Medicine, University of California, San Francisco, CA USA; Department of Cardiology, Kaiser Permanente Oakland Medical Center, 3600 Broadway, Oakland, CA 94611 USA

**Keywords:** Arrhythmia, Ambulatory monitoring, Diagnostic testing, Electrocardiography

## Abstract

**Background:**

Ambulatory electrocardiographic (ECG) monitoring is the standard to screen for high-risk arrhythmias. We evaluated the clinical utility of a novel, leadless electrode, single-patient-use ECG monitor that stores up to 14 days of a continuous recording to measure the burden and timing of potentially high-risk arrhythmias.

**Methods:**

We examined data from 122,815 long term continuous ambulatory monitors (iRhythm ZIO® Service, San Francisco) prescribed from 2011 to 2013 and categorized potentially high-risk arrhythmias into two types: (1) ventricular arrhythmias including non-sustained and sustained ventricular tachycardia and (2) bradyarrhythmias including sinus pauses >3 s, atrial fibrillation pauses >5 s, and high-grade heart block (Mobitz Type II or third-degree heart block).

**Results:**

Of 122,815 ZIO® recordings, median wear time was 9.9 (IQR 6.8–13.8) days and median analyzable time was 9.1 (IQR 6.4–13.1) days. There were 22,443 (18.3 %) with at least one episode of non-sustained ventricular tachycardia (NSVT), 238 (0.2 %) with sustained VT, 1766 (1.4 %) with a sinus pause >3 s (SP), 520 (0.4 %) with a pause during atrial fibrillation >5 s (AFP), and 1486 (1.2 %) with high-grade heart block (HGHB). Median time to first arrhythmia was 74 h (IQR 26–149 h) for NSVT, 22 h (IQR 5–73 h) for sustained VT, 22 h (IQR 7–64 h) for SP, 31 h (IQR 11–82 h) for AFP, and 40 h (SD 10–118 h) for HGHB.

**Conclusions:**

A significant percentage of potentially high-risk arrhythmias are not identified within 48-h of ambulatory ECG monitoring. Longer-term continuous ambulatory ECG monitoring provides incremental detection of these potentially clinically relevant arrhythmic events.

## Background

Ambulatory electrocardiographic (ECG) monitoring is the standard of care to screen symptomatic outpatient adults for high-risk ventricular and atrial arrhythmias [1–3]. However, there is marked variation in the technological features and patient compliance among different ECG monitoring systems [4, 5]. Traditional 24-h monitoring devices (i.e., Holter monitors) often do not detect symptomatic or clinically meaningful arrhythmias [6, 7].

Recent technological advances have allowed for higher fidelity recording and larger storage capacities that are able to capture full disclosure ECG recordings beyond the traditional 24- or 48-h monitoring periods. Furthermore, innovative device designs aim to increase patient convenience and patient compliance. Emerging evidence suggests that longer wear times yield greater arrhythmia detection in selected at-risk patients that could impact clinical decision-making and outcomes [8]. Although there has been very limited evaluation of this approach outside of detecting the presence of atrial fibrillation, longer monitoring periods are emerging as a new standard of care for selected patients.

To understand the applicability in day-to-day clinical practice, we evaluated contemporary results from a novel, long-term ambulatory ECG monitoring system to measure the burden and timing of potentially high-risk arrhythmias, including ventricular tachycardia, high-grade heart block and clinically significant pauses in atrioventricular conduction.

## Methods

### Data and study population

We analyzed data for all the ZIO® Service long-term continuous ambulatory ECG monitors (ZIO® Service, iRhythm Technologies, Inc., San Francisco, California) that were prescribed from November 2011 to December 2013 (*N* = 128,401). The ZIO® Patch is a lightweight, lead-wire free, single-patient-use ECG monitor that adheres to the left upper chest and records and stores up to 14 days of continuous, beat-to-beat ECG. Patients have the option of pressing a trigger button on the device and filling out a log to document symptomatic events during their wear duration, which allows for symptom-rhythm correlation in the ECG report. After a patient completes their 14-day recording, the ZIO® Patch is removed from the chest and mailed to iRhythm Technologies, Inc., where the up to 14-day single-channel recording is analyzed using a combination of proprietary algorithms and review by Certified Cardiac Technicians (CCT). The findings are then reported to the ordering physician in a report that includes information on several standard arrhythmias, including atrial fibrillation and flutter, ventricular tachycardia, supraventricular tachycardia, atrioventricular pauses, heart block, atrial and ventricular ectopic beats, and other identified arrhythmias. All components of the device are recycled after data downloading. Further details on the ZIO® Service and its analytic algorithms have been described previously [5, 9].

We applied standard quality control techniques to assemble a cleaned, analytic dataset of basic patient information and detailed information on detected arrhythmias. This included removing outliers that contained likely erroneous data, including records with heart rates >300 beats or <20 per minute, along with excluding patients <18 years old, records with start times outside our study dates, and records with wear-time or analyzable-time of less than 24 h. Analyzable time was calculated as the amount of time that the ECG patch was recording (enrollment period) minus the amount of time of unanalyzable ECG signal due to artifact.

### Outcomes

We categorized potentially high-risk arrhythmias into two types: (1) ventricular arrhythmias, including non-sustained and sustained ventricular tachycardia; and (2) bradyarrhythmias including sinus pauses >3 s, atrial fibrillation pauses >5 s, and high grade heart block including Mobitz Type II or third-degree heart block. Sustained ventricular tachycardia (VT) included VT that lasted greater than 30 s. Mobitz II heart block and third degree heart block were identified by the manufacturer according to their FDA-cleared algorithms and 100 % data curation and quality review by CCT’s trained in advanced arrhythmia detection. Symptomatic pauses were defined as a pause (greater than 3 s for sinus rhythm and greater than 5 s for those in atrial fibrillation) that occurred within 45 s of a patient trigger.

### Statistical analysis

All analyses were performed at the Kaiser Permanente Northern California Division of Research using SAS statistical software, version 9.3 (Cary, N.C.). Continuous variables were reported as means with standard deviations and categorical variables as frequencies and proportions. We calculated the proportion and associated 95 % confidence interval of patients with each arrhythmia overall and the cumulative yield per additional day of monitoring. We used chi-squared tests to compare the proportion of high-risk arrhythmias detected at 1-, 2-, and 7-days versus 14-days.

A research exemption was obtained from the institutional review board of the Kaiser Foundation Research Institute given that the analyses were completed on a fully de-identified dataset provided by iRhythm Technologies, Inc, and therefore no formal ethics approval was required for this study.

## Results

### Study sample and distribution of wear and analyzable time

**Fig. 1:**
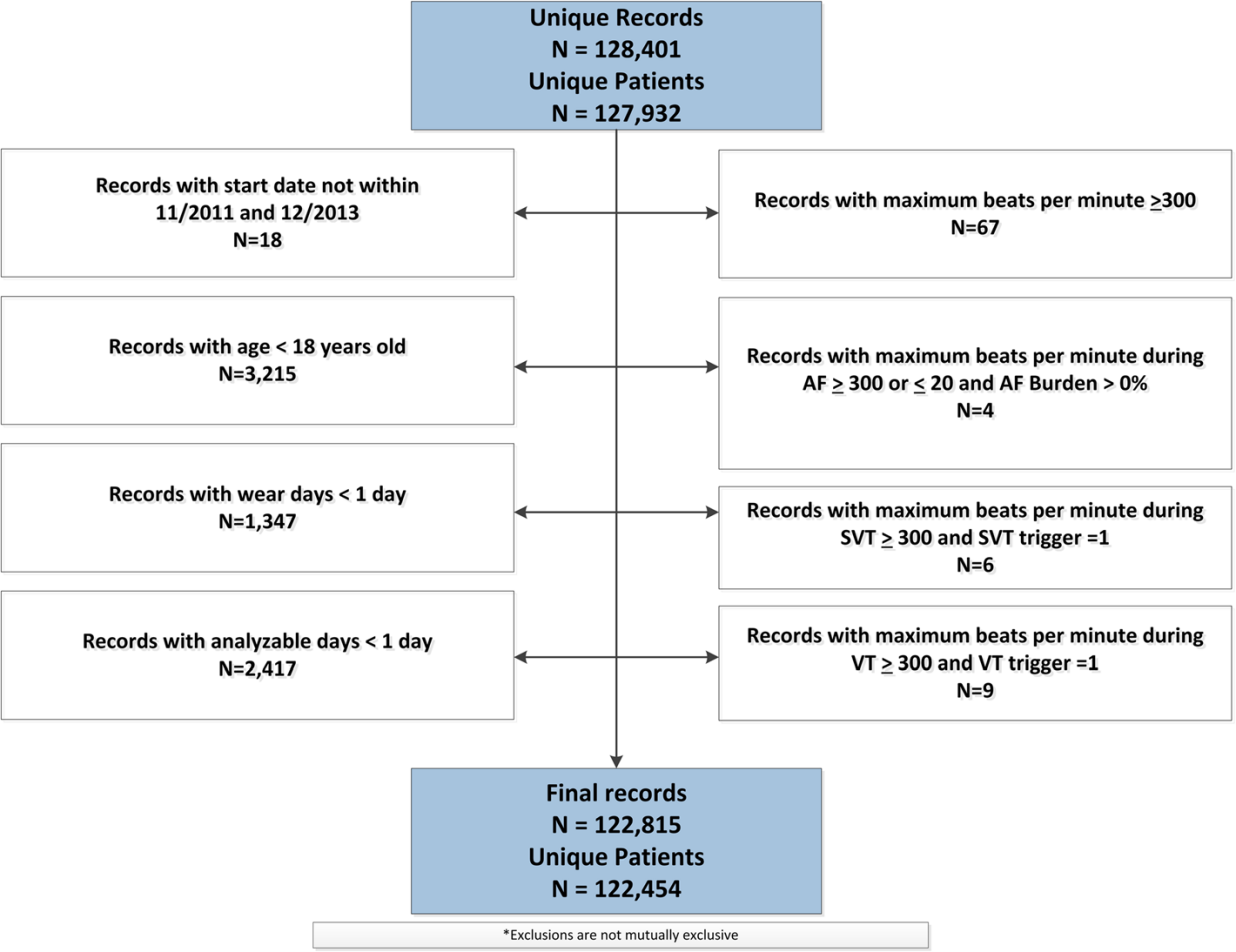
Cohort assembly

During the study period, we identified 122,815 eligible ZIO® Patch records contributed by 122,454 unique patients (Fig. 1). The overall mean wear time was 9.6 ± 4.0 days, and more than 25 % of the recorders were worn for at least 13.8 days. Analyzable time was similar, with 25 % of recorders containing greater than 13 days of analyzable time.

### Cumulative detection of potentially high-risk arrhythmias

Of the 122,815 eligible records, there were 22,443 (18 %) with nonsustained VT, 238 (0.2 %) with sustained VT, 1766 (1.4 %) with sinus pauses >3 s, 521 (0.4 %) with AF pauses >5 s, 249 (0.2 %) with symptomatic pauses and 1468 (0.4 %) with high-grade heart block (Table 1). Overall, ventricular arrhythmias were more prevalent than bradyarrhythmias, although this was driven by the large burden of episodes of nonsustained VT.

**Table 1:** Characteristics of 122,815 eligible continuous ambulatory ECG monitoring records between November 2011 and December 2013

Characteristics	Overall	Non-sustained ventricular tachycardia	Sustained ventricular tachycardia	Sinus pause	Atrial fibrillation pause	Symptomatic pause	High-grade heart block^a^
	*N* = 122,815	*N* = 22,443	*N* = 238	*N* = 1,766	*N* = 521	*N* = 249	*N* = 1,468
Age, years, N (%)							
< 65	61,170 (49.8)	7,787 (34.7)	102 (42.9)	497 (28.1)	88 (16.9)	60 (24.1)	481 (32.8)
65–79	42,469 (34.6)	9,596 (42.8)	102 (42.9)	735 (41.6)	272 (52.2)	133 (53.4)	582 (39.7)
≥ 80	19,176 (15.6)	5,060 (22.6)	34 (14.3)	534 (30.2)	161 (30.9)	56 (22.5)	405 (27.6)
Women, N (%)	65,081 (53.0)	8,316 (37.1)	59 (24.8)	698 (39.5)	238 (45.7)	131 (52.6)	571 (38.9)
Wear Days							
Mean (Standard Deviation)	9.6 (4.0)	10.8 (3.5)	10.7 (3.4)	10.7 (3.7)	11.1 (3.4)	10.9 (3.5)	10.4 (3.7)
Median (IQR)	9.9 (6.8–13.8)	12.9 (7.1–13.9)	12.1 (7.1–13.9)	12.9 (7.0–14.0)	13.0 (7.3–14.0)	12.9 (7.1–14.0)	12.1 (7.0–13.9)
Analyzable Days							
Mean (Standard Deviation)	9.2 (3.9)	10.4 (3.5)	10.1 (3.4)	10.2 (3.7)	10.5 (3.3)	10.3 (3.4)	9.9 (3.7)
Median (IQR)	9.1 (6.4–13.1)	11.8 (7.0–13.6)	11.3 (6.9–13.4)	11.8 (6.9–13.6)	12.1 (7.1–13.6)	11.7 (7.0–13.5)	10.9 (6.8–13.5)

^a^Included Mobitz II heart block and third-degree heart block

More than half (53 %) of the recorders were worn by women; but for nearly all arrhythmias except symptomatic pauses, there were more detected arrhythmias among men than women. Although nearly half (49.8 %) of the analyzed patients were for patients aged less than 65 years old, the majority of all detected arrhythmias were among patients aged 65 years or older.

### Timing of detection of potentially high-risk arrhythmias

**Fig. 2:**
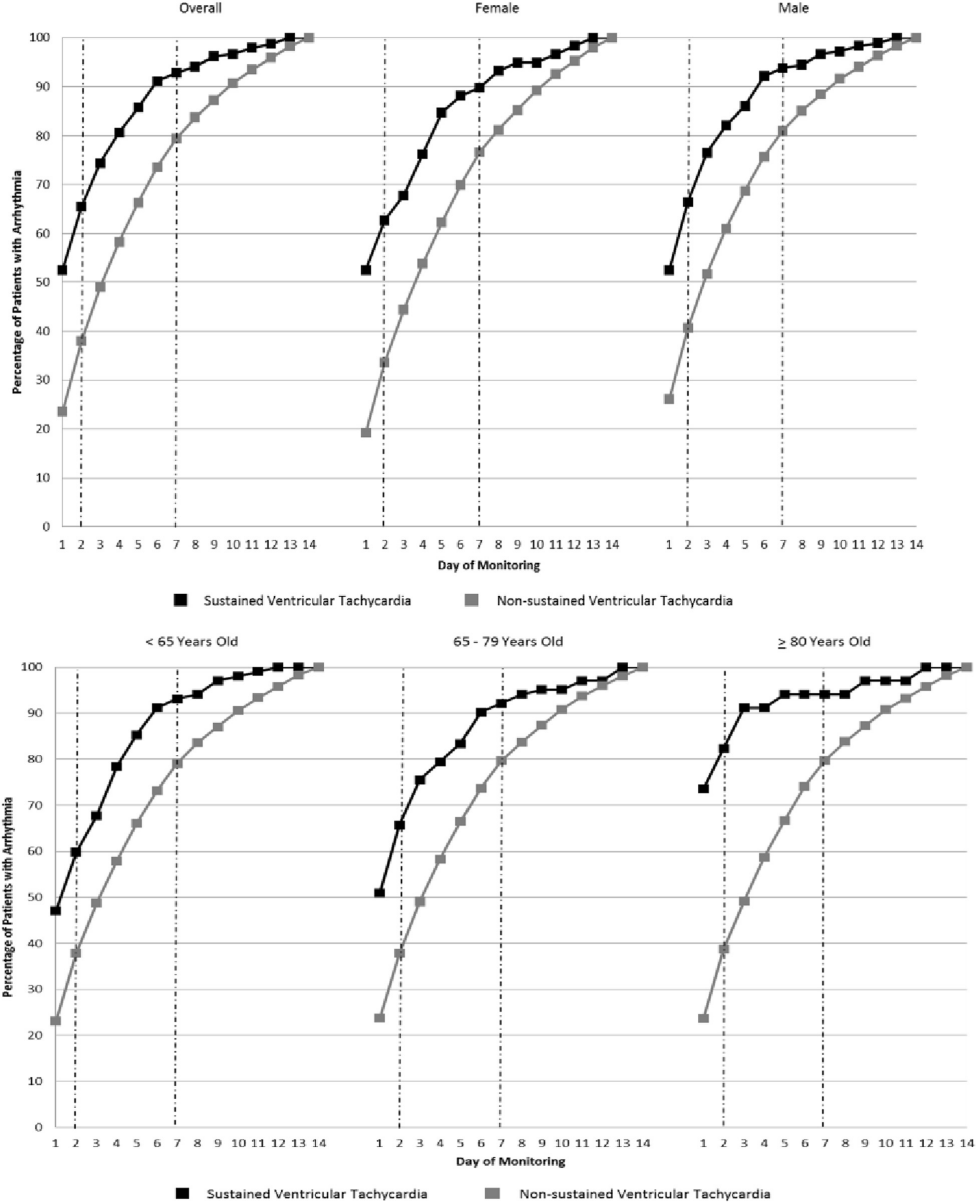
Cumulative Yield of Sustained and Non-sustained Ventricular Tachycardia. Data are shown for the overall population of patients with the arrhythmia and stratified by age and gender

For the detection of ventricular arrhythmias, there was a marked increase in arrhythmia detection over the course of the 14-day monitors (Fig. 2). For sustained VT, only 52.5 % of the total identified arrhythmias were identified at 24 h, and approximately two-thirds (65.5 %) were identified by 48 h. Most arrhythmias were identified by 7 days (92.9 %), but the additional 7 days of monitoring between 7 and 14 days yielded an additional 7.1 % of these potentially lethal arrhythmias. A similar trend was seen for the more common non-sustained VT, with 23.4, 38.0, and 79.4 % of all non-sustained VT being identified by 1, 2, and 7 days respectively. These trends were similar for both men and women and across all age ranges, although sustained VT was detected earlier among very elderly patients (aged 80 years and older).

**Fig. 3:**
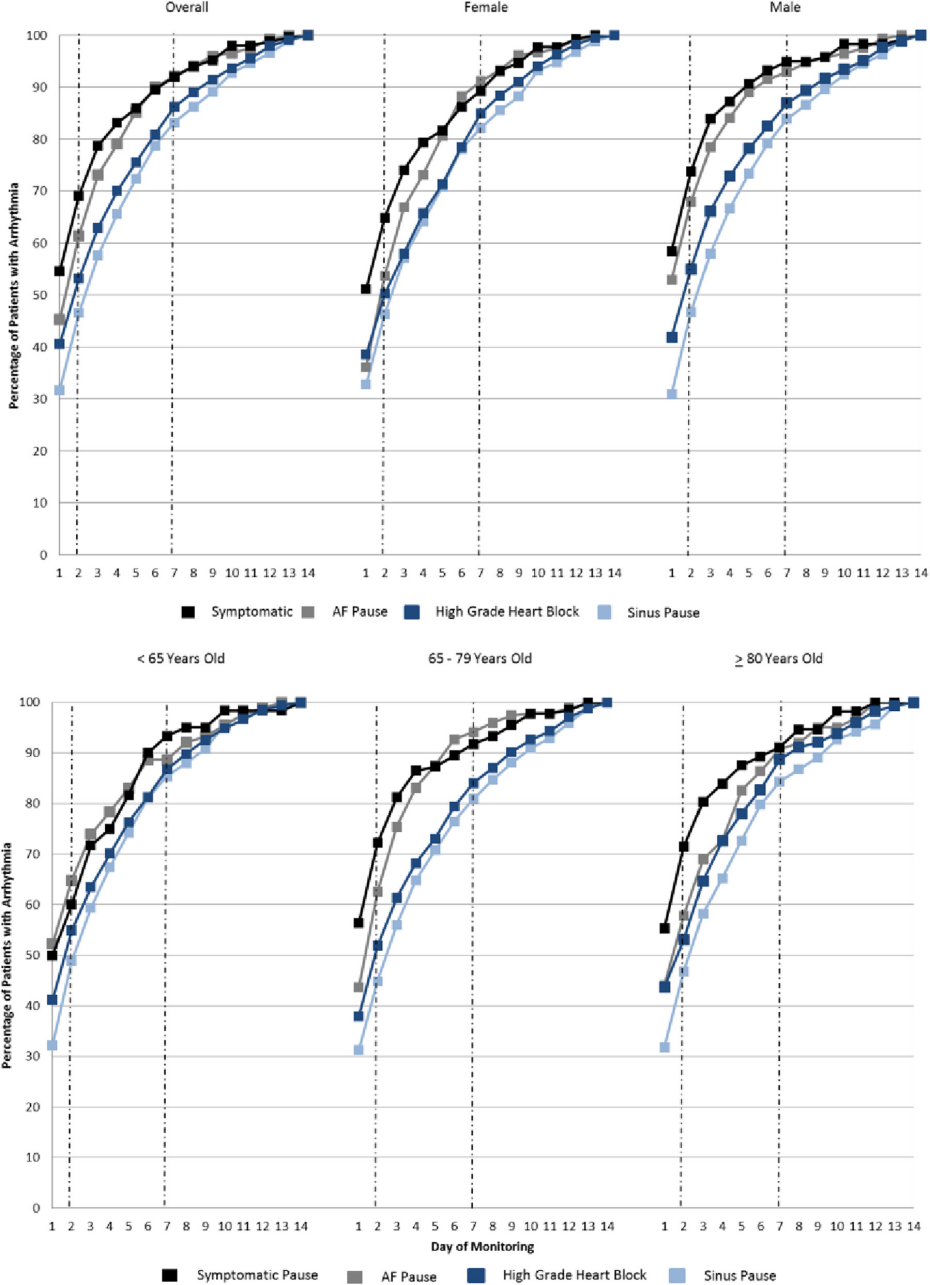
Cumulative yield of pauses and high grade heart block. Data are shown for the overall population of patients with the arrhythmia and stratified by age and gender. AF = atrial fibrillation

The detection of potentially high risk bradyarrhythmias was similarly enhanced with longer monitoring periods (Fig. 3). The difference in bradyarrhythmia yield between 1-, 2- and 7-days was substantial. For the most common bradyarrhythmia, sinus pauses >3 s (*N* = 1766), 31.7 % of the total detected arrhythmias were found within 1 day, 46.6 % within 2 days, and 83.1 % within 3 days of monitoring. Similar trends were observed for the other bradyarrhythmias, and these trends were consistent for both genders and across age categories. The diagnostic yields at 2 days versus 7 days for ventricular arrhythmias and bradyarrhythmias were significantly different from each other in the overall populations (*P* < 0.01).

## Discussion

Within a very large, contemporary study analyzing nearly 123,000 long-term continuous ambulatory cardiac monitors that were prescribed between 2011 and 2013, we found a moderate burden of potentially high-risk arrhythmias, including both ventricular arrhythmias and bradyarrhythmias. Patient compliance with extended monitoring was high, with at least 25 % of patients achieving greater than 13 days of continuous monitoring. For all arrhythmias examined, longer monitoring times significantly increased the yield of detected arrhythmias. While the gains in arrhythmia yield were particularly marked in the first 7 days of monitoring, it is notable that the gains continued to increase from days 7 to 14. The relatively high wear and analyzable time for the longer term continuous monitors suggests that outpatient ECG monitoring using this approach is feasible and can have significant yield of clinically important arrhythmias beyond atrial fibrillation. Our study examined a more recent time period than prior investigations of long-term continuous monitors [5, 9] and suggests that changes in the device technology and accumulated operator experience may have resulted in improved patient compliance.

Prior research suggests that traditional 24-h Holter monitoring is not sufficiently long enough to detect many types of arrhythmias [10–13], and recent evidence has demonstrated that longer monitoring may be useful to detect arrhythmias in high-risk patient populations, such as those with a recent history of cryptogenic ischemic stroke [8, 14], although the majority of these studies have focused primarily on finding atrial fibrillation. For example, in a registry of 239 patients who wore 30-day loop recorders after discharge for cryptogenic ischemic stroke, researchers found that 24 % of all detected cases of occult atrial fibrillation were found in the final 10 days of 30-day monitoring (i.e., between days 20 and 30) [14]. Similarly, in a larger controlled trial of a similar patient population where 24-h Holter monitoring was compared to 30-day monitoring, 17 % of all cases of atrial fibrillation were detected in the final week of monitoring [8]. Although conventional wisdom suggests that longer monitoring may be useful for detecting rarer, potentially high-risk arrhythmias, such as ventricular arrhythmias and bradyarrhythmias, there is little empirical evidence on the impact and diagnostic yield of longer continuous monitoring for other clinically meaningful arrhythmias outside of atrial fibrillation. One advantage to the studied technology compared to typical 24- or 48-h Holter monitor systems is its longer continuous wear time up to 14 days, as well as its application without any long wires attached to distant electrodes. For longer monitoring periods, loop or event recorders have typically been the preferred technology, with the main disadvantage being that recordings are only stored if they meet pre-defined algorithms or for symptomatic triggers. A post-hoc investigation of the patient’s rhythm pre-or post-event cannot be done. Implantable loop recorders are occasionally used for very rare arrhythmia events, but these have the same limitations as loop and event monitors and also require a small surgical procedure to implant the device with its attendant risks.

For ventricular arrhythmias, we found that although the majority of arrhythmias are identified in the first 7 days, a significant proportion of arrhythmias were still detected in the 7 to 14-day monitoring window. This was more pronounced for non-sustained VT than sustained VT, with more than 20 % of non-sustained VT being identified in the 7–14 day window. Although we did not have detailed clinical characteristics for our patient population, in high-risk patients, such as those with cardiomyopathy, non-sustained VT can be a high risk marker that may warrant a change in treatment such as the consideration of an implantable cardioverter-defibrillator in certain patient populations (i.e., hypertrophic cardiomyopathy). Depending upon the clinical circumstances, both nonsustained and sustained VT often support the need for further diagnostic testing, such as the evaluation for structural heart disease or for cardiac ischemia. Similarly, although potentially high-risk bradyarrhythmias were less common, if they are not appropriately identified and treated, patients may suffer significant morbidity and excess mortality. The consideration of therapeutic interventions such as permanent pacemaker implantation is recommended by the joint American College of Cardiology, American Heart Association, and Heart Rhythm Society guidelines for the high-risk bradyarrhythmias evaluated in our study [15].

Our study had certain limitations. We did not have data on any changes in clinical management or patient outcomes following monitoring, so we were unable to delineate the direct clinical impact from the detection of arrhythmias found from the monitors in our study. We did not have information on all symptomatic triggers, and thus did not analyze the proportion of all symptomatic triggers that correlate to true arrhythmias. Patient information was limited to demographic characteristics, and data were unavailable on patients’ comorbidities, which could potentially help further risk stratify patients and allow for predictive modeling to help identify those most at-risk for high-risk arrhythmias. In addition, some bradyarrhythmias, such as asymptomatic sinus and AF pauses, may occur nocturnally in normal subjects. Further, we did not validate the data on the clinical indication for the ordered monitors, and differences among providers’ thresholds for ordering the monitors could have an impact on patient selection and arrhythmia yield. Finally, while average wear time was high, at least a quarter of patients wore the device for less than 7 days (25th percentile of 6.8 days), thus artificially reducing the yield of detected arrhythmias from 7 to 14 days. Thus, the actual yield of detected arrhythmias from days 7 to 14 or monitoring may be even higher than we observed.

## Conclusion

In sum, our study suggests that longer term monitoring up to 14 days resulted in high patient compliance, and greater detection of high-risk arrhythmias than 24- or 48-h monitoring strategies. We observed similar findings across gender and age subgroups. Although the bulk of arrhythmias were detected within the first 7 days, longer-term monitoring between 7 and 14 days yielded a significant number of likely clinically meaningful, potentially high-risk arrhythmias. Future research should examine the clinical utility of improved high-risk arrhythmia detection in targeted patient groups and its impact on patient management and associated clinical outcomes.
